# Supplementary material for: Antarctic Soil and Viable Microbiota After Long-Term Storage at Constant −20 °C
Source: Biology (Basel). 2025 Feb 20;14(3):222. doi: 10.3390/biology14030222 (PMC11940283; doi:10.3390/biology14030222)
Supplement: Supplementary file 1 [file biology-14-00222-s001.zip › Supplementary S3.pdf]

Identification: *Pseudomonas graminis*

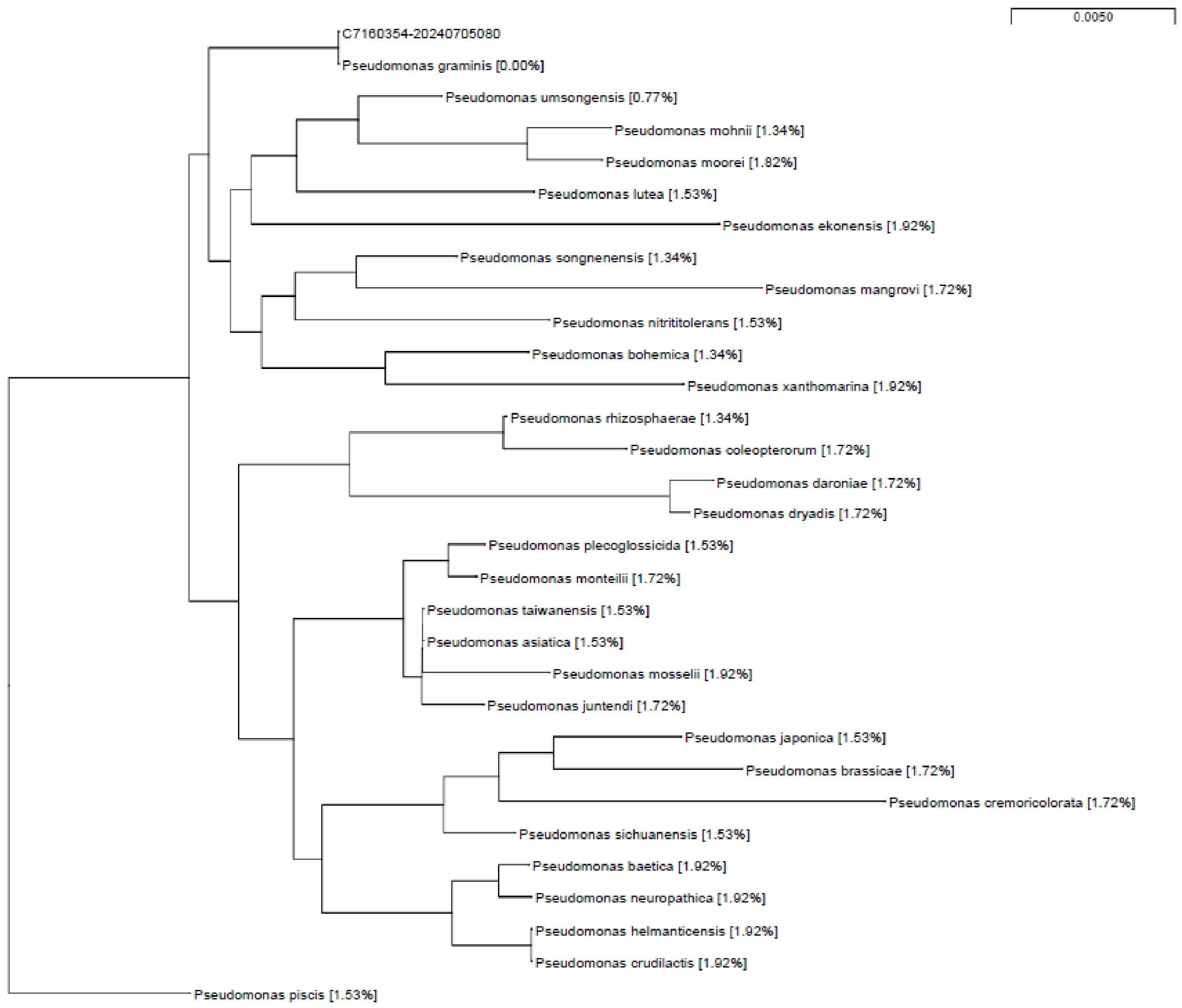

Identification: *Geomyces pannorum*

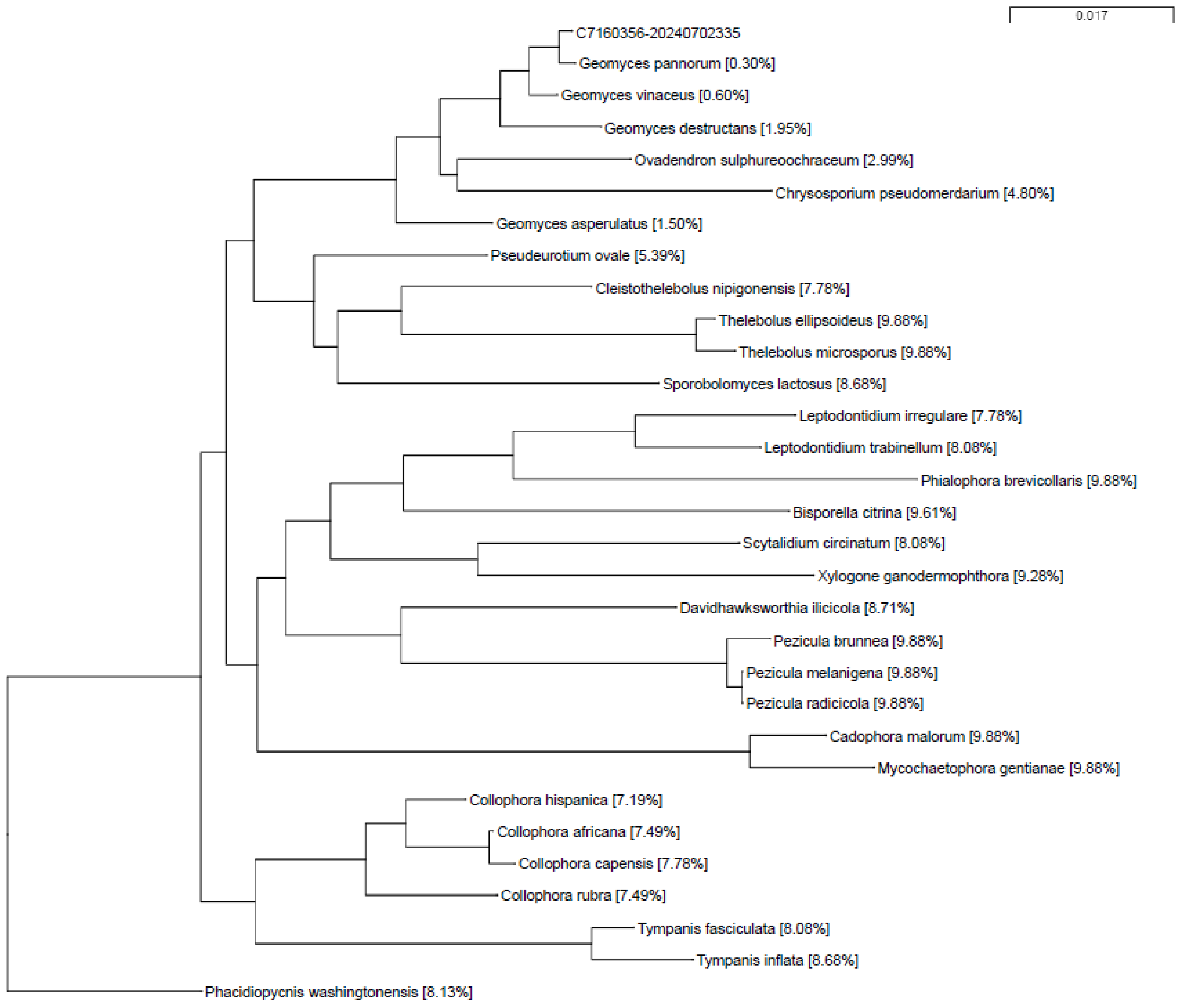

Identification: *Pseudomonas arsenicoxydans*

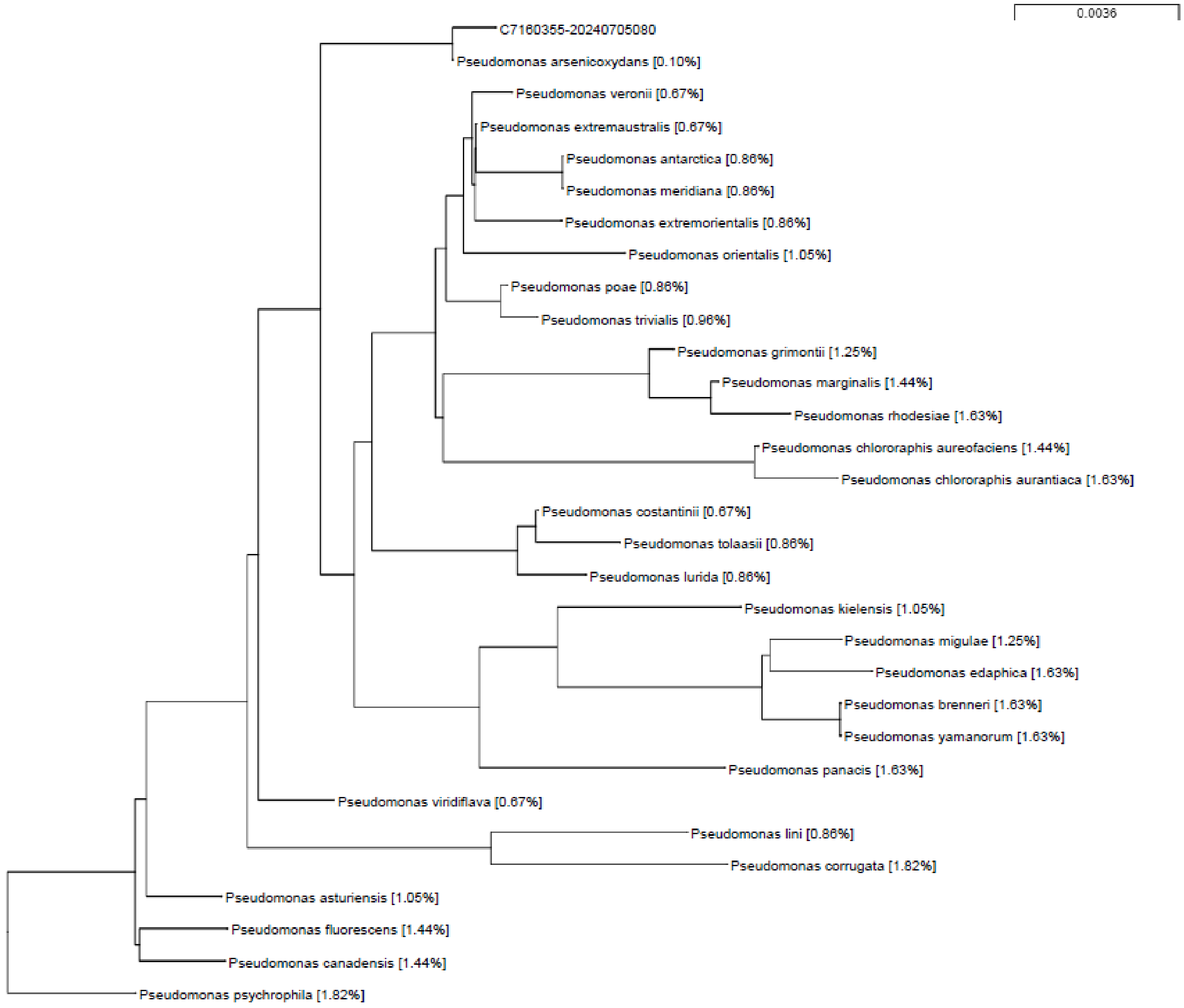

Identification: *Sporosarcina aquimarina*

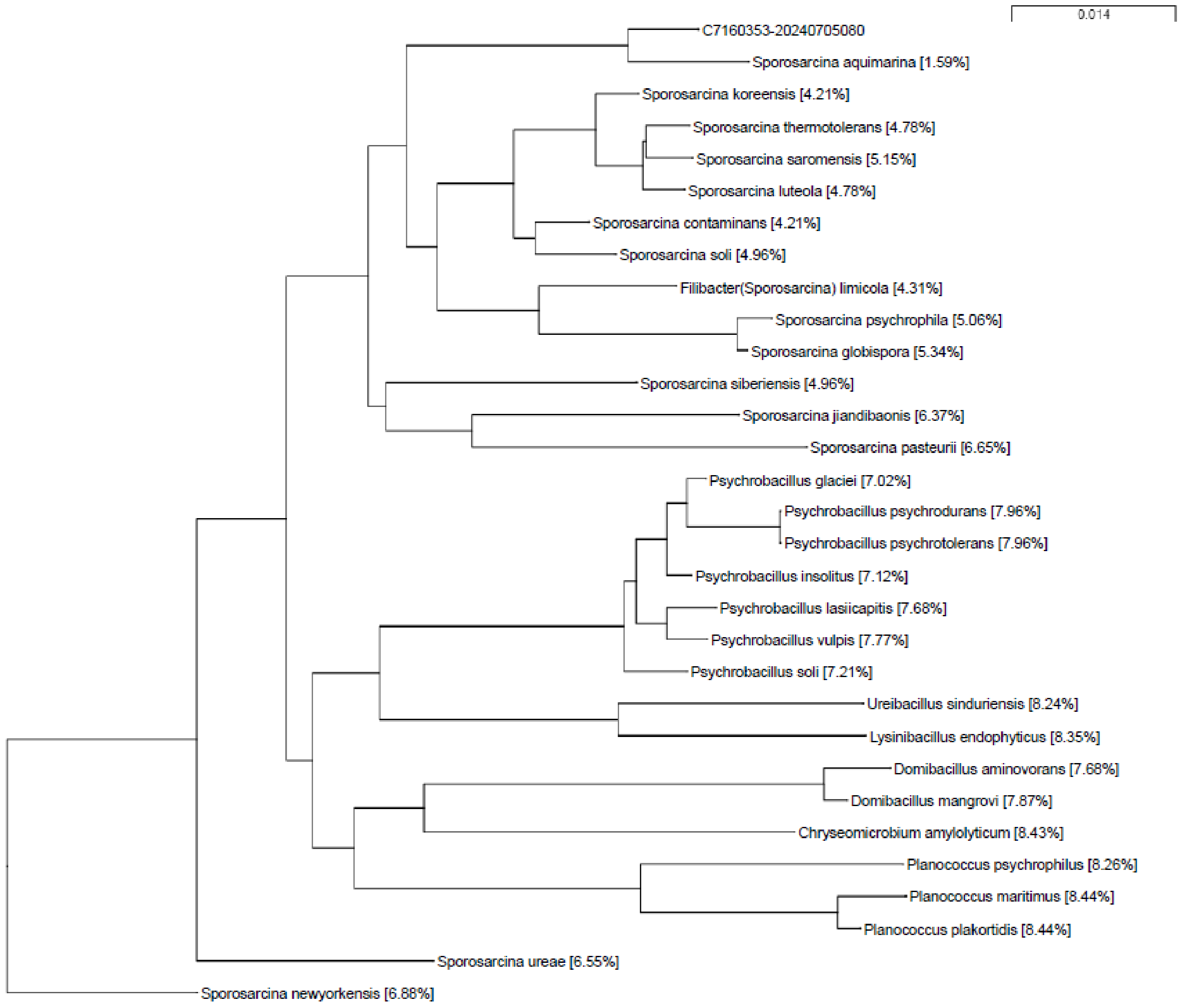

The value shown in the bracket represents the percent difference in the sequence alignment between the unknown and each individual library entry.
